# Melatonin Improves Mitochondrial Dynamics and Function in the Kidney of Zücker Diabetic Fatty Rats

**DOI:** 10.3390/jcm9092916

**Published:** 2020-09-10

**Authors:** Ahmad Agil, Meriem Chayah, Lucia Visiedo, Miguel Navarro-Alarcon, José Manuel Rodríguez Ferrer, Mohamed Tassi, Russel J. Reiter, Gumersindo Fernández-Vázquez

**Affiliations:** 1 Department of Pharmacology and Neurosciences Institute, School of Medicine, University of Granada, 18016 Granada, Spain; meriemchayah@gmail.com (M.C.); lvisiedo2@gmail.com (L.V.); 2 Biosanitary Research Institute of Granada (ibs.GRANADA), University Hospital of Granada, 18016 Granada, Spain; 3 Department of Nutrition and Bromatology, School of Pharmacy, University of Granada, 18071 Granada, Spain; nalarcon@ugr.es; 4 Department of Physiology, School of Medicine, University of Granada, 18016 Granada, Spain; jmferrer@ugr.es; 5 Service of Microscopy, CIBM, University of Granada, 18016 Granada, Spain; tassi@ugr.es; 6 Department of Cellular and Structural Biology, University of Texas Health Science at San Antonio, San Antonio, TX 78229, USA; reiter@uthscsa.edu; 7 Service of Endocrinology, La Paz Hospital, 28046 Madrid, Spain; gumersindo.fernandez@salud.madrid.org

**Keywords:** nephropathy, diabetes, obesity, melatonin, mitochondrial function/dynamic, fission/fusion, Drp1, Mfn2, Opa1, ZDF rats

## Abstract

Obesity and associated diabetes (diabesity) impair kidney mitochondrial dynamics by augmenting fission and diminishing fusion, which results in mitochondrial and renal dysfunction. Based on available evidence, the antioxidant activities of melatonin may improve impaired renal mitochondrial function in obese diabetic animals by restoring the imbalanced dynamics through inhibiting fission and promoting fusion. Male Zücker diabetic fatty (ZDF) rats and lean littermates (ZL) were orally treated either with melatonin (10 mg/kg BW/day) (M-ZDF and M-ZL) or vehicle (C-ZDF and C-ZL) for 17 weeks. Kidney function was evaluated by measurement of total urine volume, proteinuria, creatinine clearance, and assessment of kidney mitochondrial dynamics and function. C-ZDF exhibited impaired dynamics and function of kidney mitochondria in comparison to C-ZL. Melatonin improved nephropathy of ZDF rats and modulated their mitochondrial dynamics by reducing expression of Drp1 fission marker and increasing that of fusion markers, Mfn2 and Opa1. Furthermore, melatonin ameliorated mitochondrial dysfunction by increasing respiratory control index and electron transfer chain complex IV activity. In addition, it lowered mitochondrial oxidative status. Our findings show that melatonin supplementation improves nephropathy likely via modulation of the mitochondrial fission/fusion balance and function in ZDF rats.

## 1. Introduction

Mitochondrial morphology is strongly biodynamic and varies quickly in response to cellular stress or to changed metabolic demand [1]. Mitochondria are molded by continuing fusion and fission activities. The imbalance between these reverse procedures is usually harmful to mitochondrial homeostasis. Mitochondrial fission is controlled by dynamin-related protein 1 (Drp1), while mitochondrial fusion involves mitofusin 2 (Mfn2) and optic atrophy protein type 1 (OPA1) [2,3]. Indeed, a shift in this balance has been linked with mitochondrial dysfunction and implicated in several diseases, such as obesity, diabetes, and their related nephropathy (reviewed in [4,5,6,7,8]). Studies in different animal models reveal that mitochondrial impairment is characterized by dynamic imbalance, reduced oxidative phosphorylation (OXPHOS) capacity, mitophagy and creating a mitochondrial prooxidant state, which worsens the renal function in obesity and diabetes (reviewed in [9,10,11]). In fact, a rise in the phosphorylation of Drp1atserine 616 (p-Drp1ser616) exhibited a positive relationship with exaggerated fat amount and increased insulin resistance [12], and the genetic ablation of Drp1 deteriorates mitochondrial function, including the decreased activity of complexes I, III and IV [13]. Moreover, rodents with genetic mutilation of fusion markers (Mfn2 and OPA1) suppressed OXPHOS and insulin-stimulated ATP synthesis and caused a dysregulation of glucose homeostasis and insulin signaling, which leads to an impairment of the of obese-related insulin resistance and diabetes [14,15]. All these reports emphasize the functions of mitochondrial dynamics in the control of mitochondrial function, glucose metabolism and insulin signaling, and then in the progression to diabesity and its nephropathy.

The use of mitochondria-targeted agents, such as melatonin [16,17] could be an effective strategy to modulate its dynamic, oxidative, and bioenergetic deterioration, and ameliorate renal injury in obesity and diabetes. Melatonin secreted by the pineal gland [18,19] regulates circadian rhythms. It is also generated in many other tissues where it modulates mitochondrial homeostasis with antioxidant and anti-inflammatory effects [20]. Additionally, several studies, including ours, demonstrated that melatonin limits obesity, dyslipidemia, hyperglycemia, liver steatosis, systematic meta-inflammation and oxidative stress [21,22,23,24] suggesting that melatonin may have beneficial effects on kidney injury in the diabetic obese condition. In fact, two recent reports indicated that melatonin acts against diabetes and obesity-induced nephropathy [25,26]. Melatonin promotes mitochondrial fusion by increasing Mfn2 levels and cell apoptosis, with an improvement in the renal morphological damage associated with obesity in mice [26]. However, whether melatonin could improve mitochondrial dynamics by inhibiting its fission and promoting its fusion, and its implications on mitochondrial and kidney function in diabetic obese rats have never been investigated.

The ZDF rat is extensively used as a diabesity model because it recapitulates the pathogenesis and evolution of human type 2 diabetes mellitus (T2DM). Thus, it exhibits insulin resistance, and increased meta-inflammatory and prooxidative status, with hyperlipidemia and progressive nephropathy [27,28,29,30,31]. Twenty two-week-old ZDF rats exhibit a deterioration of kidney mitochondrial bioenergetics as demonstrated by diminished complex IV activity and ATP production [31]. Nephrons are rich in mitochondria. Therefore, mitochondrial dynamic imbalance and dysfunction have a key role in the progression of kidney diseases. Thus, this work determined whether chronic in vivo melatonin treatment alleviates nephropathy induced by diabesity through improvement of kidney mitochondrial dynamics and functions in diabetic obese ZDF rats.

## 2. Experimental Section

### 2.1. Chemicals

All reagents used were of the highest purity available. Melatonin was obtained from Sigma Chemicals (Madrid, Spain).

### 2.2. Animals and Experimental Protocols

The experiment was approved by the Ethical Committee of the University of Granada (Granada, Spain) according to the European Union guidelines. The permit project number is 4 09-2016-CEEA.

### 2.3. Experimental Protocol

Male ZDF rats (fa/fa *n* = 16) and male lean littermates (ZL, fa/- *n* = 16) were obtained at the age of 5 weeks. This study was carried out in accordance with the European Union guidelines for animal care and protection. Animals were maintained on Purina 5008 rat chow (protein 23%, fat 6.5%, carbohydrates 58.5%, fiber 4%, and ash 8%; Charles River) and housed 2 per clear plastic cage in a climate-controlled room at 28–30 °C and 30–40% relative humidity, with a 12-h dark/light cycle (lights on at 07:00 h). In the first week after arrival, the animals were acclimated to room conditions, and water intake was recorded. Then, both ZL and ZDF rats were subdivided into two groups: animals treated for 17 weeks with melatonin in drinking water (melatonin-treated, M-ZDF and M-ZL) and vehicle-treated controls (C-ZDF and C-ZL). Melatonin was dissolved in a minimum volume of absolute ethanol and diluted in the drinking water to yield a dose of 10 mg/kg body weight-BW-/day, with a final concentration of 0.066% (*w*/*v*) ethanol. Water intake and body weight were recorded every two days. Fresh melatonin and vehicle solutions were prepared every two days, and the melatonin dose was adjusted to the body weight throughout the study period. Water bottles were covered with aluminum foil to protect from light, and the drinking fluid was changed every two days.

### 2.4. Clearance Studies: Measurement of Urinary and Serum Parameters

After a 17-week administration period, rats were housed in metabolic cages for 3 whole days and given chow and water ad libitum. Urine samples for the last 2 days were used for measurement of urinary parameters and total urine volume. Proteinuria was measured by using the Bradford method (Bio-Rad Protein Assay kit, Bio-Rad Laboratories, Hercules, CA, USA). Creatinine (in serum and urine) were quantified with standard kits (Roche Diagnostics) using a Hitachi 912 E analyzer.

### 2.5. Mitochondria Preparation

At the end of the treatment period, animals were anesthetized with sodium thiobarbital (thiopental) and sacrificed. Mitochondria were isolated from kidney tissues by serial centrifugation [32]. Tissues were removed, excised, washed with cold saline, and homogenized in isolation medium (10 mm Tris, 250 mm sucrose, 0.5 mm Na_2_EDTA, and 1 g/L free fatty acid BSA, pH 7.4, 4 °C) with a Teflon pestle.

The homogenate was centrifuged at 1000× *g* for 10 min at 4 °C, and the supernatant was centrifuged again at 15,000× *g* for 20 min at 4 °C. The resultant pellet was resuspended in 1 mL of isolation medium without BSA, and an aliquot was frozen for protein measurement. The remaining mitochondrial suspension was centrifuged at 15,000× *g* for 20 min at 4 °C and resuspended in 1 mL of respiration buffer (20 mm HEPES, 0.5 mm EGTA, 3 mm MgCl_2_, 20 mm taurine, 10 mm KH_2_PO_4_, 200 mm sucrose, and 1 g/L free fatty acid BSA). The mitochondrial suspension was kept on ice for 10–15 min. To allow for the rearrangement of the membranes, the mitochondrial suspension was incubated on ice for 10–15 min before starting the measurements.

Protein concentration was measured by the Bradford method [33] using bovine serum albumin as a standard. To standardize the procedure, protein quantification was performed using samples of equal weight and volume. Tissues were cut into small pieces, and a 0.2–0.3 g sample was subjected to the mitochondrial isolation procedure. 0.2 mL of mitochondrial suspension, prepared as described above, was used to determine protein concentration.

### 2.6. Western Blotting for Mitochondrial Dynamic Proteins

100 mg protein isolated from kidney mitochondria was used for western blotting of Drp1, Mfn2, and OPA1 factors. Primary antibodies against rat Drp1 (sc-271583), Mfn2 (sc-515647), and OPA1 (sc-393296) (1:2000) were purchased from Sigma-Aldrich (U6382) and mouse β-actin antibody (sc-81178) (Santa Cruz Biotechnology; SC-81178; Santa Cruz, CA, USA) was used to measure β-actin as a loading control and used as previously described [24]. Protein samples were resolved by SDS-PAGE (sodium dodecyl sulphate polyacrylamide gel electrophoresis). The gels for immunoblot analyses were transferred to a nitrocellulose membrane (Bio-Rad Trans-Blot SD, Bio-Rad Laboratories). After blocking for 1 h in blocking solution (5% non-fat milk, TBS) the membranes were incubated overnight at 4 °C with proper primary antibodies and their controls diluted according to the manufacturers’ instructions. Then, the unbound primary antibodies were removed and the membranes were incubated for 1 h at room temperature with horse radish peroxidase (HRP)-conjugated antibodies. Proteins were visualized by enhanced chemiluminescence method (ECL kit, Healthcare Life Sciences). The density of the band was analyzed by ImageJ software.

### 2.7. Mitochondrial Respirometry

Mitochondrial respiration was evaluated using the high-resolution Oroboros oxygraph-2k equipment (Oroboros Instruments, Innsbruck, Austria) consisting of a two-chamber respirometer with a Peltier thermostat and electromagnetic stirrers [34]. Analysis was performed in 2 mL of respiration medium. The medium was previously equilibrated in each chamber with air at 30 °C and stirred at 750 rpm until a stable signal at air saturation was obtained. A final concentration of 0.2–0.3 mg/mL fresh proteins of isolated mitochondria in the respiratory buffer was used for the experiments. The mitochondria were suspended in respiration buffer supplemented with glutamate (5 mm)/malate (2.5 mm) or with succinate (5 mm) in the presence of rotenone as energizing substrates. Oxygen flux (JO2) was recorded at 30 °C in a constantly stirred oxygraph vessel after successive additions of 1 mm ADP (state 3ADP or OXPHOS capacity) and 0.75 mm oligomycin as ATPase inhibitor (state 4 or leak respiration). Results were expressed as pmol of oxygen consumed/min.mg protein for each respiratory state. Measurements were taken at 0.2-s intervals for 15–20 min and recorded using a computer-driven data acquisition system (DatLab, Innsbruck, Austria). The respiratory acceptor control ratio (RCR) (the ratio of state 3 to state 4) was used as a general measure of mitochondrial function [35].

### 2.8. Spectrophotometric Assay of Respiratory Chain Complex

Complex IV activity (nmol oxidized cytochrome c/min/mg protein) was determined in a medium containing 75 mM potassium-phosphate pH 7.4 at 25 °C. The reaction was started by the addition of cytochrome C previously reduced with sodium borohydride [36]. The activity was measured as the disappearance of reduced cytochrome C at 550 nm.

### 2.9. Determination of Mitochondrial Nitrites

The method involves the use of the Griess diazotization reaction to spectrophotometrically detect nitrite formed by the spontaneous oxidation of NO under physiological conditions [37]. This determination was carried out after the mitochondrial isolation, using a Griess Reagent Kit (G-7921; Molecular Probes, Life Tecnologies, Madrid, Spain), as described in manufacturer’s instructions. The optimum measurement wavelength is 548 nm.

### 2.10. Mitochondrial Activity of SOD

The superoxide dismutase (SOD, both Mn and Cu/Zn) activity was measured using a SOD Assay Kit-WST (19160; Sigma-Aldrich, Buchs, Switzerland), after the mitochondrial isolation, following the kit instruction. Results were expressed as inhibition rate percentage (SOD activity). The minimum detectable amount for SOD activity was 0.001 U/mL.

### 2.11. Statistical Analysis

Data were expressed as means ± S.E.M. Means were compared among groups by using a two-way ANOVA followed by the Tukey’s test for comparison between groups. SPSS version 15 for Windows (SPSS, Michigan, IL, USA) was used for analyses. A *p* < 0.05 was considered statistically significant, and levels of significance were labeled on the figures as follows: *** *p* < 0.001; ** *p* < 0.01; * *p* < 0.05, and ^###^ *p* < 0.001; ^##^ *p* < 0.01; ^#^ *p* < 0.05.

## 3. Results

### 3.1. Renal Function Assessment

Table 1 shows data on total urine volume (mL/day), proteinuria (mg/day), and creatinine clearance rate (ml/min). Compared with lean rats, obese animals exhibited altered kidney function as evidenced by 8.3-fold increase of (85.8 ± 6 mL/day in ZDF rats vs. 10.3 ± 1.3 in ZL rats, *p* < 0.001), 19.5-fold increased proteinuria (94 ± 18 mg/day in ZDF vs. 4.8 ± 0.9 in ZL, *p* < 0.001), and 22% reduced creatinine clearance levels (2.5 ± 0.2 mL/min in ZDF vs. 3.2 ± 0.1 in ZL, *p* < 0.05). Melatonin supplementation of ZDF rats partially reversed the renal dysfunction by 35% decrease of total urine volume (*p* < 0.001) a 29% decrease of proteinuria (*p* < 0.001), and 12% increase of creatinine clearance levels (*p* < 0.05).

### 3.2. Western Blotting Drp1, Mfn2, and OPA1 Expression Results

**Figure 1:**
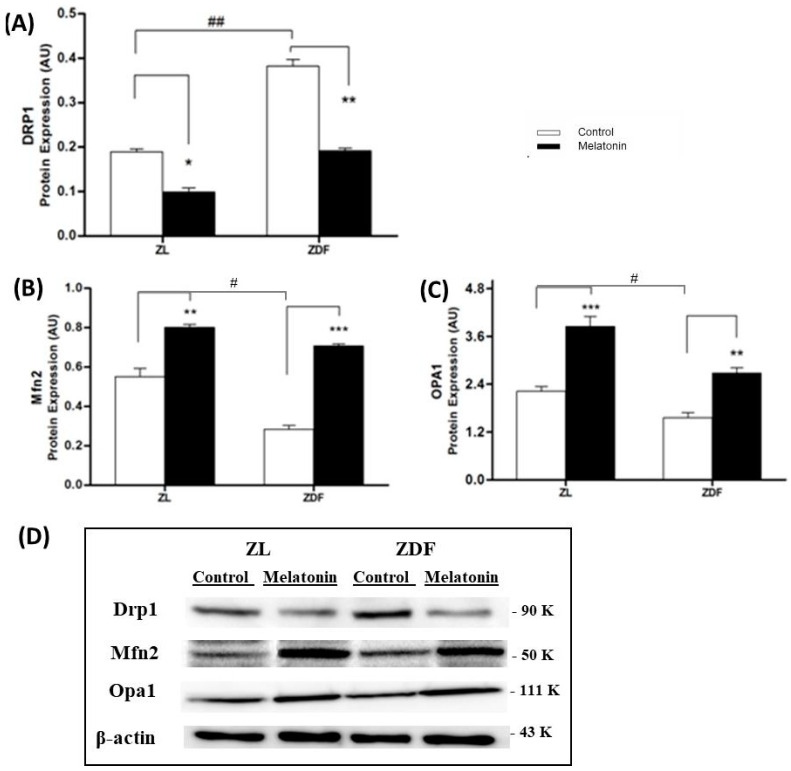
Effect of melatonin treatment on kidney mitochondria dynamic markers protein levels: (**A**) Drp1, Fission expression, and (**B**) Mfn2 and (**C**) Opa1, fusion levels in mitochondria isolated from different treated groups by western blot. (**D**) Representative western blots are shown. Values are the means ± S.E.M. ZL, Zücker lean rats (*n* = 8); ZL-M (ZL – melatonin treated) (*n* = 8), ZDF, Zücker diabetic fatty rats (*n* = 8), and Zücker Diabetic Fatty-M (ZDF- melatonin treated) (*n* = 8) rats, after 16 weeks of treatment. Superscript letters refer to significant difference measured using two-way ANOVA (* *p* < 0.05, ** *p* < 0.01, *** *p* < 0.001 M-ZDF compared with C-ZDF, and M-ZL compared with C-ZL rats; ^#^ *p* < 0.05, ^##^ *p* < 0.01 C-ZDF compared with C-ZL rats).

The fusion and fission balance is key for correct kidney mitochondrial functionality and thereby for basic cellular events like generation of ATP and reactive oxygen species, among others. Accordingly, possible effects of melatonin on mitochondrial dynamics were investigated using western blot (Figure 1). The fission is mediated by Drp1. This marker is two-fold higher in C-ZDF rats compared to C-ZL (0.39 ± 0.01 and 0.18 ± 0.006 respectively; *p* < 0.01) indicating an imbalance towards mitochondrial fragmentation in obese diabetic (C-ZDF) rats (Figure 1A,C). Melatonin treatment of ZDF rats completely restores Drp1 expression to levels in lean animals (Figure 1A,C). Meanwhile, fusion is operated by Mfn2 and by OPA1 proteins. As shown in Figure 1B,C, obese-diabetic animals have lower expressions of Mfn2 (40% lower, *p* < 0.05) and Opa1 (30% lower, than lean rats. Melatonin treatment increased fusion markers expression in both ZDF and ZL animals. Mfn2 exhibited a 2.5-fold raise (*p* < 0.001) and Opa1 a 1.7-fold raise (*p* < 0.05) in treated ZDF rats compared with control.

### 3.3. Mitochondrial Respirometry

**Figure 2:**
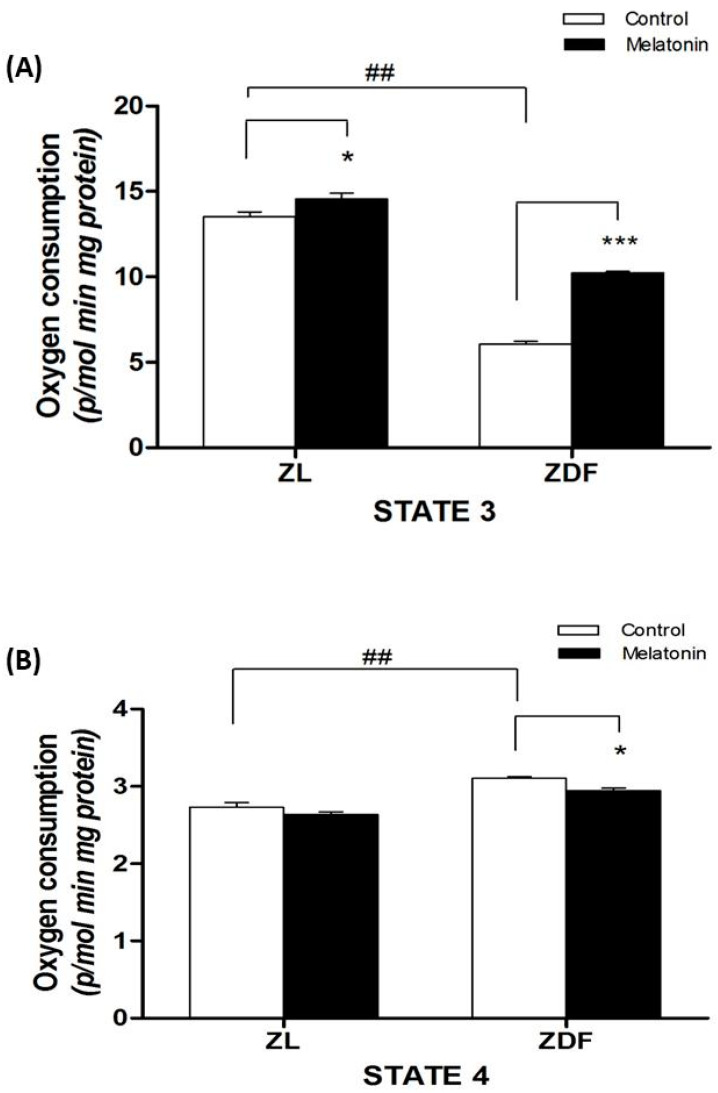
Effect of melatonin treatment on the respiratory states in isolated mitochondria from renal tissue. (**A**) The upper panel shows the state 3 (oxygen flux while producing ATP in response to ADP pulses in the presence of substrates). (**B**) The lower panel documents the state 4 or leak respiration (oxygen flux in the absence of ATP synthesis). Glutamate/malate was used as respiratory substrates. Values are the means ± S.E.M. ZL, Zücker lean rats (*n* = 8); ZL-M (ZL – melatonin treated) (*n* = 8), ZDF, Zücker diabetic fatty rats (*n* = 8), and Zücker diabetic fatty-M (ZDF- melatonin treated) (*n* = 8) rats, after 16 weeks of treatment. (*** *p* < 0.001, * *p* < 0.05 M-ZDF compared with C-ZDF, and M-ZL compared with C-ZL rats; ^##^ *p* < 0.01 C-ZDF compared with C-ZL rats).

The optimal function of the mitochondria is essential for energy metabolism. Thus, we studied if melatonin supplementation affects mitochondrial respiration in isolated mitochondria from kidney tissue, in both animal phenotypes. As showed in Figure 2A, mitochondria isolated from obese-diabetic ZDF animals showed a lower state 3 respiration compared with ZL littermates (6.0 ± 0.2 in ZDF vs. 13.3 ± 0.3 in ZL, *p* < 0.001). The melatonin increased state 3 respiration in the mitochondria of both lean (13.3 ± 0.3 in C-ZL vs. 14.8 ± 0.3 in M-ZL rats, *p* < 0.05) and fat diabetic animals (6.0 ± 0.2 in C-ZDF vs. 10.3 ± 0.1 in M-ZDF rats, *p* < 0.001) (Figure 2). In addition, mitochondria from renal tissue of diabetic obese ZDF rats exhibited a 15 % increase of leaking respiration (state 4) compared with ZL lean rats (2.6 ± 0.2 in ZDF vs. 2.2 ± 0.3 in ZL, *p* < 0.01). Melatonin treatment reduced leak respiration by 8 % in ZDF (2.4 ± 0.03 in M-ZDF vs. 2.6 ± 0.04 in C-ZDF, *p* < 0.05) (Figure 2B).

**Figure 3:**
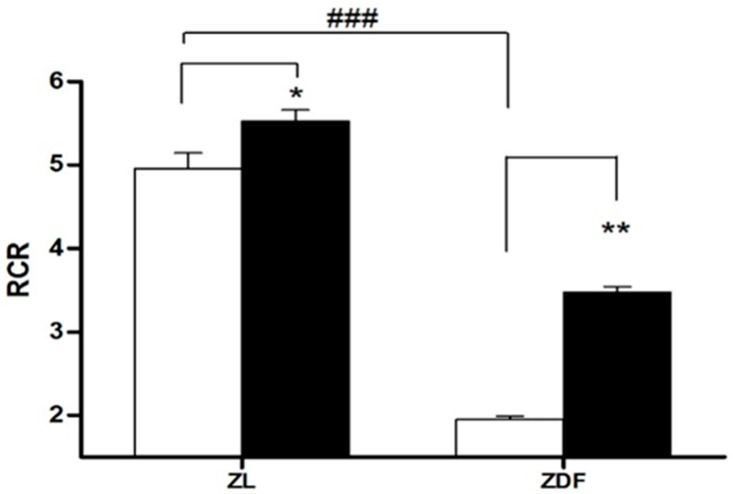
Respiratory control ratio (RCR) in isolated mitochondria from renal tissue. RCR is defined as the ratio state 3/state 4. Values are the means ± S.E.M. ZL, Zücker lean rats; ZDF, Zücker diabetic fatty rats. Superscript letters refer to significant difference measured using two-way ANOVA (* *p* < 0.05, ** *p* < 0.001 M-ZDF compared with C-ZDF, and M-ZL compared with C-ZL rats; ^###^ *p* < 0.01 C-ZDF compared with C-ZL rats).

Figure 3 summarizes data on the RCR. As expected from data of states 3 and 4, mitochondria isolated from obese ZDF have a RCR 60% lower than ZL controls (1.93 ± 0.05 μmol/mg protein in ZDF vs. 4.9 ± 0.020 in C-ZL rats, *p* < 0.01). The melatonin treatment restores by 44% the mitochondrial respiration RCR in ZDF rats (from 1.97 ± 0.05 in C-ZDF to 3.59 ± 0.07 in M-ZDF rats, *p* < 0.01). In all performed experiments, the data for RCR were >1.97 showing both an excellent mitochondrial preparation and a proper coupling of isolated mitochondria [38].

### 3.4. Mitochondrial Complex IV Activity Results

**Figure 4:**
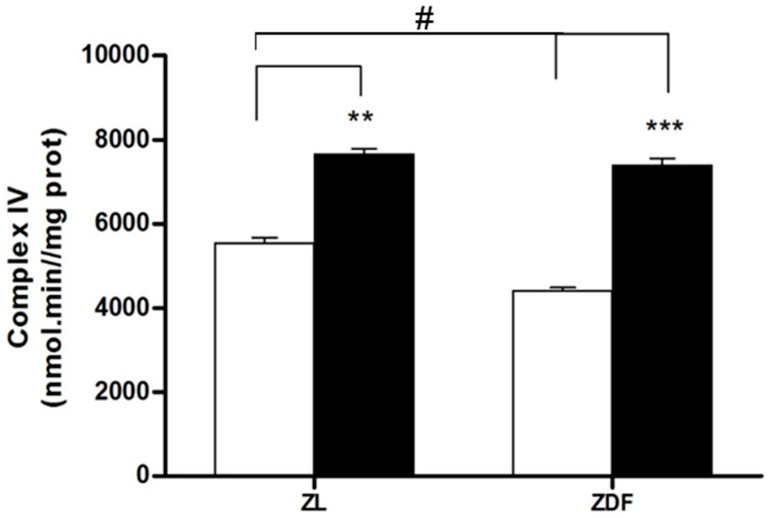
Activity of complex IV of the electron transport chain (ETC) in renal mitochondria isolated from different treatment groups. Values are the means ± S.E.M. ZL, Zücker lean rats; ZDF, Zücker diabetic fatty rats. Values are the means ± S.E.M. ZL, Zücker lean rats; ZDF, Zücker diabetic fatty rats. Superscript letters refer to significant difference measured using two-way ANOVA (** *p* < 0.01, *** *p* < 0.001 M-ZDF compared with C-ZDF, and M-ZL compared with C-ZL rats; ^#^ *p* < 0.01 C-ZDF compared with C-ZL rats).

The mitochondrial complex IV activity, a prototypical component and the final protein complex of the ETC (inner membrane), was examined (Figure 4). The activity of complex IV in kidney tissue decreased by 25% in fatty diabetic animals (from 56.1 ± 5.2 μmol/mg protein in C-ZL to 42.2 ± 2.6 in C-ZDF; *p* < 0.05). The melatonin supplementation clearly raised complex IV activity in obese (by 42%, from 42.2 ± 2.6 in C-ZDF to 73.3 ± 2.5 μmol/mg protein in M-ZDF rats, *p* < 0.001) and in lean animals (by 27% from 56.1 ± 5.2 in C-ZL to 77.5 ± 0.8 μmol/mg protein in M-ZL rats, *p* < 0.01).

### 3.5. Mitochondrial Oxidative Status

One of the key roles of melatonin in mitochondria is associated with its antioxidant activity.

#### 3.5.1. Nitrite Levels

**Figure 5:**
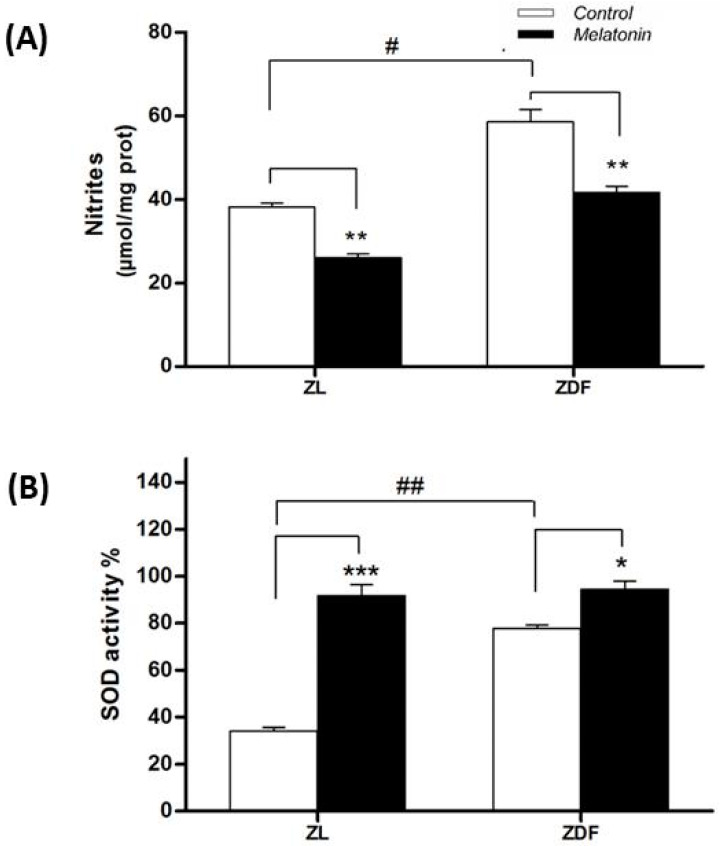
Effect of melatonin on (**A**) nitrite levels and (**B**) superoxide dismutase (SOD) activity in mitochondria isolated from renal tissue. Values are the means ± S.E.M. ZL, Zücker lean rats; ZDF, Zücker diabetic fatty rats. Superscript letters refer to significant difference measured using two-way ANOVA (* *p* < 0.05, ** *p* < 0.01, *** *p* < 0.001 M-ZDF compared with C-ZDF, and M-ZL compared with C-ZL rats; ^#^ *p* < 0.05, ^##^ *p* < 0.01 C-ZDF compared with C-ZL rats).

Nitrite levels in kidney tissue raised in fatty diabetic rats (from 38.1 ± 1 μmol/mg protein in C-ZL to 58.5 ± 2.9 in C-ZDF, *p* < 0.01) (Figure 5A). Melatonin supplementation clearly diminished nitrites levels in renal mitochondria of ZDF rats (from 58.5 ± 2.9 to 41.6 ± 1.5 μmol/mg protein in ZDF rats, *p* < 0.01).

#### 3.5.2. Superoxide Dismutase Activity

The SOD activity levels in kidney tissue were raised in control obese rats (from 34.1 ± 1.5 μmol/mg protein in C-ZL to 77.7 ± 1.6 in C-ZDF; *p* < 0.01) (Figure 5B). Melatonin-supplemented rats showed greater SOD activity in renal mitochondria of lean rats (from 34.1 ± 1.5 to 91.7 ± 4.7; *p* < 0.001) and diabetic obese ones (from 77.7 ± 1.6 to 94.3 ± 3.6; *p* < 0.05) (Figure 5B).

## 4. Discussion

Herein, we report the beneficial action of melatonin on nephropathy in obese diabetic animals. As far as we know, this present study shows that this compound improves renal mitochondrial dynamic balance and functions obese diabetic ZDF rats, as evidenced by modulation of fission/fusion dynamics in both opposing directions, inhibiting mitochondrial fission and promoting mitochondrial fusion, enhanced organelle respiration, increased complex IV activity, reduced nitrite production, and improved anti-oxidative capacity.

Obese ZDF rats exhibited kidney dysfunction as evidenced by alteration of several markers, including increased urinary excretion, a non-selective marker, increased proteinuria, a conventionally marker with frequent false negative reading test and decreased creatinine clearance levels, the best integral marker of kidney failure and glomerular filtration rate. These results are consistent with other reports [39,40]. Melatonin treatment of diabetic obese ZDF rats improves renal dysfunction as evidenced by a reduced total urine volume (30%) and proteinuria (71%) and an elevated creatinine clearance (16%). These melatonin effects are in agreement with results obtained in other experimental and clinical conditions (reviewed in [41]). Melatonin treatment of obese (ob/ob) mice reduced serum creatinine [26], and urine albumin/creatinine ratio, despite the fact that serum urea and creatinine levels remained unaffected upon short treatment of 4 weeks [25]. The beneficial effects of melatonin on diabesity-induced nephropathy could be due to restoration of mitochondrial dynamic and functional integrity. Alternatively, it could be attributed to the improvement of the metabolic complications of obesity, like diabetes (glucotoxicity), lipidemia disorder (lipotoxicity), hypertension, and liver dysfunction. Moreover, melatonin mitigation of systemic pro-inflammatory state and oxidative stress, could account for these benefits in diabetic obese nephropathy [21,22,23,24]. Despite this, we cannot state if the action of melatonin supplementation in the amelioration of kidney mitochondrial dynamic balance/functions in ZDF rats is directly or indirectly related with multiple factors above mentioned (fasting glucose level, obesity, hypertension, dyslipidemia, etc.).

In the present study, we observe prominent altered mitochondrial dynamic and functional damage in kidneys of ZDF rats. Mitochondria from kidney cells of ZDF animals showed strongly greater fission expression for the marker Drp1 and parallel lower fusion expression Mfn2 and Opa1 than their lean littermates. This result is in agreement with growing evidence suggesting that obesity and diabetes impair mitochondrial dynamic regulation in several tissues reviewed in [11]. Thus, a fission process is favored in mitochondria from cells exposed to nutrient-rich environment, as occurs in obesity or diabetes, contributing to mitochondrial hurt, cell injury, and death [5]. Excessive and prolonged accumulation of fat leads to a rise in free fatty acids (FFAs) in the plasma and an accumulation of lipids within the cells, resulting in overproduction of acyl coenzyme A and consequent increase in ceramides and diacylglycerol. The aforementioned compounds directly alter the mitochondrial dynamic processes by increasing the levels of Drap1 (fission) and decreasing those of Mfn2 and Opa1 (fusion) marker levels [42,43]. Moreover, a study in ischemic brain has shown that hyperglycemia of T2DM produced a fragmented state, increasing Drp1 and decreasing Mfn2 and Opa1 levels, with alterations in the regulation of mitochondrial bioenergy [44]. Moreover, in the metabolically stressed ischemic mice kidney, Mfn2 deficiency caused a severe mitochondrial fragmentation [45]. The amelioration of the mitochondrial imbalance of the kidney tissue was one of the most important impacts of melatonin supplementation in this study. Melatonin exhibited a dual benefit on mitochondrial dynamics, decreasing Drp1 expression, a marker of fission, and enhancing expression of Mfn2 and Opa1, markers of fusion. This result is supported by a report which demonstrates that melatonin increases Mfn2 in renal convoluted tubules in mice ob/ob [25]. Consequently, the regulation of mitochondrial dynamics could be a direct or indirect target for the recovery of the side effects associated with the condition of diabesity. One possible explanation is that melatonin inhibits the drp1 translocation into the mitochondria, preventing the fission process [46]. In addition, as a part of this dynamic, this indolamine inhibits drp1-mediated fission in diabetic heart and in hyperglycemic retinal conditions [47,48].

Obesity and diabetes are associated with increased ROS production, dynamic imbalance, and mitochondrial dysfunction [10,11]. The cumulating of lesions in the mitochondria due to excessive fission dynamics also alters the ETC which eventually leads to an increase in the generation of ROS [35]. These enhanced ROS disrupt the mitochondrial dynamic, leading to its dysfunction. The present work shows dysfunction in the mitochondria of the kidney in ZDF rats as evidenced by a strong worsening of OXPHOS capacity vs. ZL animals. This was noted as a decline in the state-3 and an increase in the state-4, both leading to a final decline in the RCR index (state-3/state-4). The melatonin administration enhanced the RCR of renal cell mitochondria of diabetic obese ZDF rats through both, a marked increment (58%) in state 3 of respiration and a decrease of proton leakage. Our group has previously reported that melatonin treatment improved mitochondrial liver function, including an increased rate of respiratory control (RCR), complex IV activity, and ATP production slowing down steatohepatitis and insulin resistance associated with diabesity in ZDF rats [22,24]. In this study, we noted a 25% decrease of renal mitochondrial oxidase complex IV activity in control obese (C-ZDF) rats vs. lean (C-ZL) animals. The melatonin supplementation of obese diabetic (M-ZDF) rats restores complex IV activity to similar values as those of M-ZL animals. This finding is in line with our earlier publications demonstrating elevated mitochondrial complexes I and IV activities in brown adipose tissues (BAT) of ZDF rats upon melatonin treatment [49].

Hyperglycemia (glucotoxicity) and hyperlipidemia (lipotoxicity) raise the ETC suppliers which can deplete the respiratory complexes resulting in their inhibition [50,51,52]. This inhibition in turn increases the dissipation of electrons with the consequent increase in the generation of ROS, which ultimately causes cellular damage by altering membrane structure and function through lipid peroxidation reactions [53]. Thus, ROS induce cellular injury in the renal cells [54]. Additionally, a decrease in the expression of OXPHOS genes has been indicated in humans with T2DM [55].

The production of reactive nitrogen species (RNS) increases in diseases like diabetes and obesity, which aggravates mitochondrial damage expressed by increased nitroxidative injury [37], as demonstrated in the mitochondrial liver cells in the same animal obese diabetic model (ZDF) vs. lean animals [24]. As expected, our findings showed that melatonin supplementation reduced the generation of nitrites and increased SOD activity in the renal mitochondria of the two rat phenotypes. The ability of this hormone to reduce oxidative stress in renal mitochondria is in line to other reports in different tissues [56,57,58,59,60,61,62,63,64,65] which also indicate the mitochondria as a therapeutic target for the antioxidant actions of this indolamine. In summary, melatonin therapy improves overall mitochondrial dysfunction and altered mitochondrial dynamics observed in the kidney of obese diabetic condition as others reported in other conditions in renal damage [66,67]. Our findings reinforce a statement made by others that melatonin supplementation could be useful for improvement in stable outpatient renal transplant recipients [68].

## 5. Conclusions

This study has demonstrated that melatonin supplementation enhances kidney function in diabetic obese rats through the improvement of kidney mitochondrial dynamics by inhibiting fission and promoting fusion as well as amelioration of mitochondrial physiology. This finding may encourage future studies to support the melatonin usefulness in the treatment of diabetic nephropathy.

## Figures and Tables

**Table 1:** Effects of melatonin on urinary and serum parameters indicators of nephropathy (urinary flow, proteinuria, and creatinine clearance) on ZL, Zücker lean rats (*n* =8); ZL-M (ZL—melatonin treated) (*n* = 8), ZDF, Zücker diabetic fatty rats (*n* = 8), and Zücker diabetic fatty-M (ZDF-melatonin treated) (*n* = 8) rats, after 16 weeks of treatment.

	ZL-C	ZL-M	ZDF-C	ZDF-M
Total urine volume (mL/day)	10.3 ± 1.3	10.4 ± 1.3	85.8 ± 6 ^###^	55.9 ± 8 ***
Proteinuria (mg/day)	4.8 ± 0.9	4.9 ± 0.8	94 ± 18 ^###^	67 ± 8 ***
Creatinine clearance (mL/min)	3.2 ± 0.1	3.2 ± 0.2	2.5 ± 0.2 ^###^	2.9 ± 0.2 *

Superscript letters refer to significant difference measured using two-way ANOVA (* *p* < 0.05, *** *p* < 0.001 M-ZDF compared with C-ZDF rats; ^###^ *p* < 0.001 C-ZDF compared with C-ZL rats).
